# Rational Design of Disulfide Bridges in *Bb*PETase^CD^ for Enhancing the Enzymatic Performance in PET Degradation

**DOI:** 10.3390/molecules28083528

**Published:** 2023-04-17

**Authors:** Dongjian Huang, Lin Zhang, Yan Sun

**Affiliations:** 1 Department of Biochemical Engineering, School of Chemical Engineering and Technology, Tianjin University, Tianjin 300350, China; huangdongjian@tju.edu.cn (D.H.); linzhang@tju.edu.cn (L.Z.); 2 Key Laboratory of Systems Bioengineering and Frontiers Science Center for Synthetic Biology (Ministry of Education), Tianjin University, Tianjin 300350, China

**Keywords:** *Bb*PETase^CD^, disulfide bridge, PET degradation, rational design

## Abstract

Polyethylene terephthalate (PET) is one of the most prevalent transparent thermoplastics. It is commonly utilized due to its low cost and high durability. With the massive accumulation of waste PET, however, serious environmental pollution has become a global problem. Compared to traditional chemical degradation, biodegradation of PET catalyzed by PET hydrolase (PETase) is more environmentally friendly and energy-efficient. *Bb*PETase^CD^ from the *Burkholderiales* bacterium is a PETase that shows favorable properties for application in the biodegradation of PET. To enhance the enzymatic performance of this enzyme, this work focuses on the rational design of disulfide bridges in *Bb*PETase^CD^. We utilized two computational algorithms to predict the probable disulfide-bridge mutations in *Bb*PETase^CD^, and five variants were acquired from the computations. Among these, the N364C/D418C variant with one additional disulfide bond showed higher expression than the wild-type enzyme (WT) and the best enzymatic performance. The melting temperature (*T_m_*) of the N364C/D418C variant presented an increase of 14.8 °C over that of WT (56.5 °C), indicating that the additional disulfide bond significantly raised the thermodynamic stability of the enzyme. Kinetic experiments at different temperatures also demonstrated the thermal stability increase of the variant. The variant also showed significantly increased activity over WT when using bis(hydroxyethyl) terephthalate (BHET) as the substrate. More remarkably, the N364C/D418C variant exhibited approximately an 11-fold increase over the WT enzyme in the long-term (14 days) degradation of PET films. The results prove that the rationally designed disulfide bond significantly improved the enzymatic performance of the enzyme for PET degradation.

## 1. Introduction

Plastic products are widely used due to their light weight, easy manufacturing, and high durability [1]. The annual production capacity of plastic products is about 350 million tons [2]. However, only around 9% of plastic trash is recycled, while the remainder is burnt or disposed of in landfills [3]. Plastic waste will form microplastics (<5 mm) after crushing and weathering. Under the influence of ocean circulation, microplastics have almost spread all over the world [4,5], resulting in serious environmental pollution. As one of the most common transparent thermoplastics, polyethylene terephthalate (PET) is widely used in beverage bottles, food packaging, and other fields [6,7]. However, it is estimated that in the natural environment, PET will take 300 to 450 years to degrade [8].

Traditional chemical degradation of PET includes glycolysis [9], alcoholysis [10], and so on. The chemical degradation process often requires high temperatures and pressure and will be accompanied by the production of some pollutants [11]. In contrast, the biodegradation of PET by enzymes is more energy-efficient and environmentally friendly, and a variety of PET hydrolases have been identified, such as leaf–branch compost cutinase (LCC), *Humicola insolens* cutinase (*Hi*C), *Thermobifida fusca* cutinase (*Tf*C), and *Saccharomonospora viridis* AHK 190 cutinase (Cut190) [12,13,14]. It is noted that these PET hydrolases have high optimum reaction temperature and energy requirements. Yoshida et al. [15] discovered a bacterium that can hydrolyze PET. *Is*PETase produced by the bacteria can hydrolyze PET into small molecules at moderate temperature. In addition, several PETase-like enzymes were determined through phylogenetic tree analysis, such as the *Rg*PETase from *Rhizobacter gummiphilus* [16], the *Pb*PETase from *Polyangium brachysporum*, and the *Bb*PETase from *Burkholderiales bacterium* [17]. Of these, *Bb*PETase differs from other type IIb enzymes in having an extra 143 amino acids (AAs) at the *N*-terminus. The segment was renamed *Bb*PETase^AND^, and the core domain of this enzyme was called *Bb*PETase^CD^. Although *Bb*PETase^AND^ provided higher thermal stability for the enzyme, it may limit enzyme performance by interfering with the enzyme’s interaction with the substrate PET [17]. The melting temperature (*T_m_*) of *Bb*PETase is about 6 °C higher than that of *Is*PETase, indicating its better thermal stability. Nonetheless, its stability and activity are still insufficient to satisfy the demands of practical applications.

A variety of enzyme engineering techniques have been used to improve the activity and stability of PETase, and the rational design of disulfide bridges is a reasonable scheme for improvement. Disulfide bonds primarily stabilize proteins by improving the rigidity of the protein structure and decreasing the entropy of the protein’s unfolded state [18]. Researchers improved the stability of some enzymes by computer-aided design to introduce disulfide bridges. Gihaz et al. [19] obtained a variant of lipase from *Geobacillus stearothermophilus* T6 by the computational design with DbD and MODIP programs, which had higher *T_m_* value and significantly higher activity than the wild-type enzyme. Bashirova et al. [20] enhanced the structural rigidity of endoglucanase by the rational design of disulfide-bridge mutations through the BioLuminate program, and the half-life of the obtained variants were significantly increased.

In this study, we introduced disulfide bridges in *Bb*PETase^CD^ by computer-aided design to improve the stability and activity of the enzyme. The structure of *Bb*PETase^CD^ was predicted and analyzed using DbD2 (http://cptweb.cpt.wayne.edu/DbD2/index.php, accessed on 25 November 2022) [21] in conjunction with DSDBASE-MODIP(http://caps.ncbs.res.in/iws/modip.html, accessed on 25 November 2022) [22,23]. SWISS-MODEL homologous modeling (https://swissmodel.expasy.org/interactive, accessed on 26 November 2022) [24] was used to screen out mutant combinations that could correctly form disulfide bridges. We finally obtained P207C/D280C, A209C/R283C, and N364C/D418C variants through prediction and screening, and then obtained P207C/D280C/N364C/D418C and A209C/R283C/N364C/D418C variants through combining the mutations. These enzymes were extensively characterized to find the best variant.

## 2. Results and Discussion

### 2.1. Rational Design of Disulfide Bridges and Protein Expression

We predicted the structure of *Bb*PETase^CD^ using DbD2 and the DSDBASE-MODIP program, and obtained 25 pairs versus 84 pairs of mutation combinations, respectively. After removing two pairs of natural disulfide bonds and taking the intersection, nineteen pairs of mutant combinations were obtained. After screening according to the parameters or criteria mentioned in Section 3.2, P207C/D280C, A209C/R283C, and N364C/D418C variants were obtained for subsequent experiments. The distance between P207 and A209 and the distance between D280 and R283 are shown in Figure S1. In consideration of the short distance between P207C/D280C and A209C/R283C, these two mutations were not used together in further combination of the mutations to avoid possible steric hindrance. By combining the P207C/D280C variant or A209C/R283C variant with the N364C/D418C variant, the P207C/D280C/N364C/D418C and A209C/R283C/N364C/D418C variants were obtained. The location of the disulfide bridge in the enzyme is depicted in Figure S2, where the natural disulfide bridge is marked in orange and the introduced disulfide bridge in purple. The active site of *Bb*PETase^CD^ is marked in red.

The expression of wild-type enzyme and variants was then determined. Only the N364C/D418C variant showed an expression level comparable to the wild type (Table S2), while the expression levels of the remaining variants were quite low. In the purification using affinity chromatography (Figure S3), only the *Bb*PETase^CD^ and N364C/D418C variant showed obvious elution peak. We speculate that the mutation site of the P207C/D280C and A209C/R283C variants were close to the *N*-terminal of the enzyme after removing the extra *N*-terminal domain of the enzyme [17]. It made the protein more prone to misfolding and led to low soluble expression, significantly reducing the apparent expression level of these variants.

The formation of disulfide bridge in enzyme was then examined using the protocol described in Section 3.5. The results are shown in Table 1. It can be seen that not all enzymes correctly formed disulfide bridges. The four variants with low expression had low proportion of disulfide bridges. This also supported our suspicion that introducing disulfide bridges near the protein’s *N*-terminal would reduce protein expression. Furthermore, not all disulfide bridges of the *Bb*PETase^CD^ formed upon expression, which could be attributed to the *E*. *coli* Rosetta gami-B (DE3) strain. Expression of proteins with disulfide bridges by the *E*. *coli* Rosetta gami-B (DE3) strain may be not very effective [25]. Therefore, it may adversely affect the correct formation of disulfide bridges in *Bb*PETase^CD^ and its variants. The sulfate–polyacrylamide gel electrophoresis (SDS-PAGE) results of *Bb*PETase^CD^ and its variants are shown in Figure S4a. Minor bands near the target band were observed, which was considered as the enzyme without correct formation of disulfide bridges. Moreover, Figure S4b,c demonstrates that the bacterial lysates and precipitates of all the four variants except the N364C/D418C variant contain darker bands around 30 kDa, while their supernatants obtained after centrifugation have only very light bands around 30 kDa. This indicates that all the four variants produced many inclusion bodies during the expression. This result confirmed our conjecture that the four variants were less expressed in soluble form.

### 2.2. Activity and Stability of BbPETase^CD^ and Its Variants

**Figure 1:**
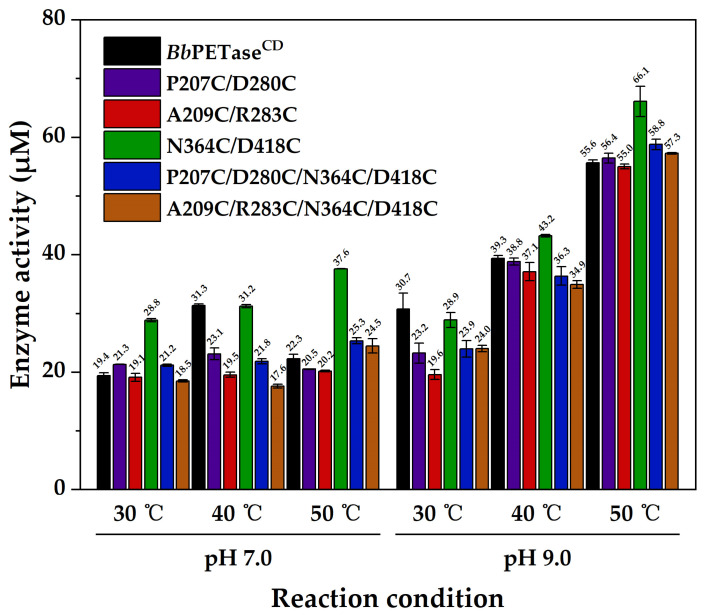
Activity of *Bb*PETase^CD^ and its variants using BHET as substrate. The final concentration of enzyme was 50 nM. The final concentration of BHET was 200 mg/L.

The activity of *Bb*PETase^CD^ and its variants was evaluated using bis(Hydroxyethyl) Terephthalate (BHET) as substrate. The N364C/D418C variant had the highest activity in most cases, and no significant difference was observed between the other variants (Figure 1). At pH 9.0, the activity increased with increasing temperature. Meanwhile, at each temperature tested (30, 40, 50 °C), the activity at pH 9.0 was higher than that at pH 7.0, indicating that pH 9.0 is more suitable for the reaction. At pH 7.0, the activity of *Bb*PETase^CD^ increased from 30 °C to 40 °C, and then decreased when the temperature increased to 50 °C, indicating the poor stability of *Bb*PETase^CD^ at high temperature.

**Figure 2:**
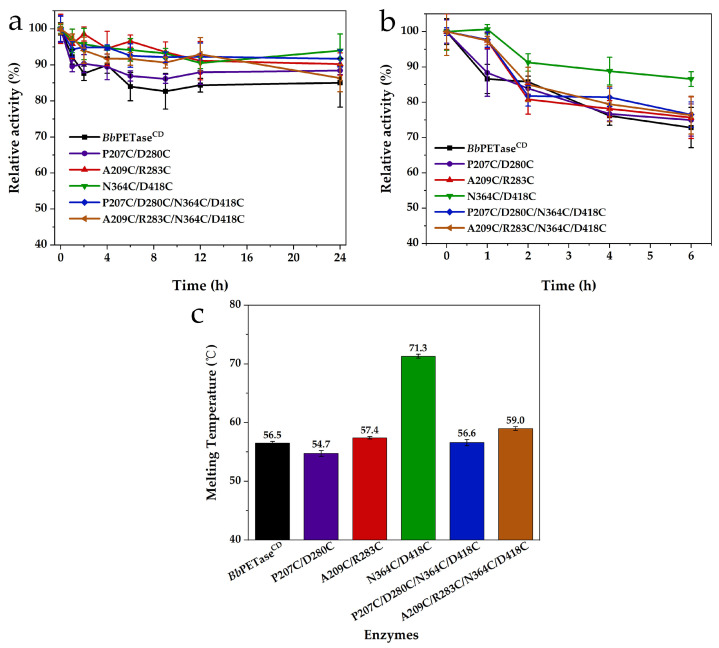
Residual activity of *Bb*PETase^CD^ and its variants against BHET after incubation at 40 °C (**a**), and 50 °C (**b**). The reaction temperature after incubation was 50 °C. The reaction buffer was basic reaction buffer at pH 9.0. The final concentration of enzyme was 50 nM. The final concentration of BHET was 200 mg/L. The *T_m_* value of *Bb*PETase^CD^ and its variants (**c**).

To evaluate the thermal stability, these enzymes were then incubated at 50 °C for 1, 2, 4, and 6 h following by the examination of residual activity. Meanwhile, these enzymes were incubated for 1, 2, 4, 6, 9, 12, and 24 h at 40 °C (the temperature used for the long reaction time in PET degradation experiments). The residual activity of these enzymes decreased as the temperature of the incubation increased, but the magnitude of the decrease varied. At both 40 °C and 50 °C, the residual activity of the N364C/D418C variant was higher than that of the *Bb*PETase^CD^ (Figure 2a,b). The optimal reaction pH for *Bb*PETase^CD^ and its variants is mostly alkaline. Therefore, these enzymes are more able to maintain their activity at pH 9.0. The *T_m_* values of these enzymes were obtained by CD (Figure S5), which showed that the *T_m_* value of the N364C/D418C variant was 71.3 °C (Figure 2c), significantly higher than that of the *Bb*PETase^CD^ (56.5 °C) and other variants. Compared to the *T_m_* value of *Bb*PETase^CD^, the P207C/D280C variant exhibited a lower *T_m_* value, while the other variants had a slightly higher *T_m_* value. However, when the incubation temperature was 40 °C, the residual activity of the N364C/D418C variant did not show significantly higher value than that of the other variants. It indicated that the addition of disulfide bridges in the N364C/D418C variant significantly improved the thermodynamic stability of the enzyme, but had no obvious effect on its kinetic stability.

### 2.3. Activity of BbPETase^CD^ and Its Variants for PET Film Degradation

**Figure 3:**
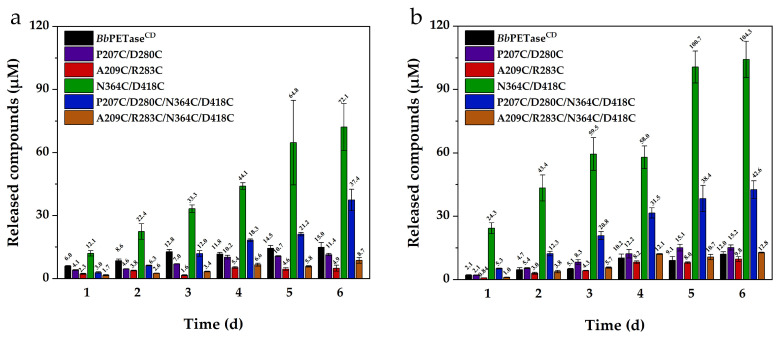
Hydrolytic activity of *Bb*PETase^CD^ and its variants on PET films at 30 °C (**a**) and 40 °C (**b**). The final concentration of the enzyme was 200 nM. Two PET films of 6 mm diameter were placed in each centrifuge tube as substrates.

The activity of *Bb*PETase^CD^ and its variants for PET degradation were tested using the protocol described in Section 3.9. The differential scanning calorimetry (DSC) curve of the substrate, Nongfu Spring PET mineral water bottle is shown in Figure S6. For this reaction, 30 °C and 40 °C was tested; 50 °C was not used because the activity of these enzymes decreased significantly when the hydrolysis reaction of PET was performed at this temperature. At 30 °C, the reaction time for test was 6 days. At 40 °C, the reaction time was extended to 14 days. The degradation activity of *Bb*PETase^CD^ and its variants on PET at 40 °C was higher than that at 30 °C. Therefore, the reaction time was extended to 14 days at 40 °C. It can be seen that the hydrolytic activity of N364C/D418C variant on PET film was significantly higher than that of *Bb*PETase^CD^ (Figure 3a,b). The total amount of soluble products released was up to 11-fold than that obtained using *Bb*PETase^CD^. Furthermore, the PET hydrolysis activity of *Bb*PETase^CD^ at 30 °C was always higher than that at 40 °C, which further indicated its low thermal stability. N364C/D418C and P207C/D280C/N364C/D418C variants, on the other hand, displayed significantly higher reactivity at 40 °C than *Bb*PETase^CD^ and other variants. For the remaining three variants, the activity level was comparable to or much lower than that of *Bb*PETase^CD^. We speculated that the addition of disulfide bridges to the P207C/D280C and A209C/R283C variants caused most of the enzyme to be degraded during expression due to misfolding and that a minor portion of the undegraded enzyme was also negatively affected. This is reflected in their significantly lower PET hydrolysis activity than that of *Bb*PETase^CD^ (Figure 3). Moreover, the activity of P207C/D280C/N364C/D418C and A209C/R283C/N364C/D418C variants were significantly lower than that of N364C/D418C variant. This further indicated that the mutation sites of P270C/D280C and A209C/R283C were unfavorable to the *Bb*PETase^CD^.

**Figure 4:**
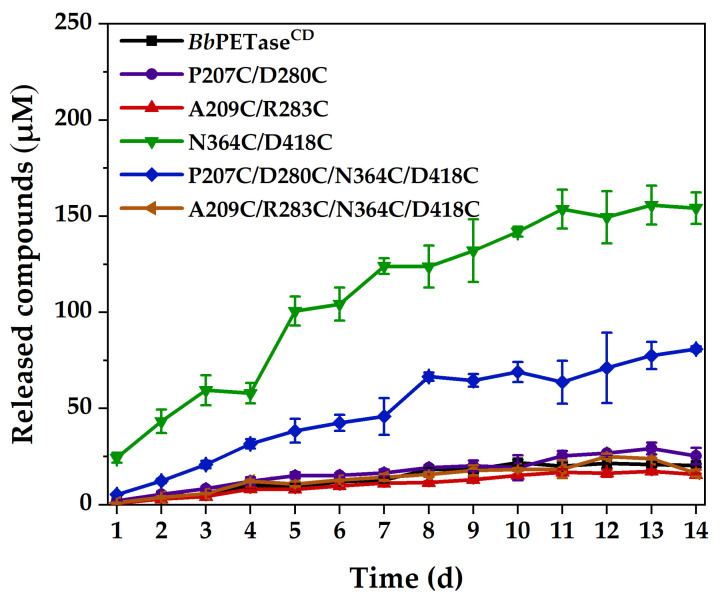
Hydrolytic activity of *Bb*PETase^CD^ and its variants on PET films at 40 °C. The final concentration of the enzyme was 200 nM. Two PET films of 6 mm diameter were placed in each reaction centrifuge tube as substrates.

Following that, these PET films’ incubation time was extended to 14 days at 40 °C, and the comparative activity is shown in Figure 4. The activities of N364C/D418C and P207C/D280C/N364C/D418C variants were significantly higher than those of *Bb*PETase^CD^ and other variants. The activities of *Bb*PETase^CD^ and other variants did not differ significantly. Li et al. [26] showed that the introduction of disulfide bonds in the same region could reduce the local unfolding rate of the enzyme, which in turn would enhance the structural rigidity of the region. The N364C/D418C mutation in this study was closer to the two natural disulfide bonds and the active site of the enzyme than the other two mutations. It is considered that the introduction of the N364C/D418C mutation increased the rigidity of the loop region. In addition, the disulfide bond might change the whole structure of the enzyme to some extent (though the change was not appreciable in Figure S2), resulting in an enlarged substrate binding pocket for the enzyme, which facilitated the binding of the enzyme to BHET or PET. Furthermore, all the enzymes were almost completely inactivated at 11 days because the soluble products almost stopped increasing after that. These PET films were washed at 14 days, and their surfaces were examined by scanning electron microscopy (SEM) for traces of enzymatic erosion, as shown in Figure S7. The surface of the PET film after incubation with the N364C/D418C variant was very rough, with obvious degradation marks. The surface of the PET film showed obvious erosion marks after incubation with the P207C/D280C/N364C/D418C variant, but the roughness was lower than that of the N364C/D418C variant. These PET films’ surfaces, on the other hand, showed only very shallow fish-scale erosion marks after incubation with the *Bb*PETase^CD^ and other variants, which is consistent with the results of PET film degradation activity.

### 2.4. Circular Dichroism (CD) and Fluorescence Analysis

To investigate the structural changes in *Bb*PETase^CD^ and its variants, these enzymes were characterized using CD and fluorescence. The CD spectra of *Bb*PETase^CD^ and its variants are shown in Figure S8a. The overall trends of the curves were similar, except for the ellipticity peak. Significant differences were observed in their secondary structures (Figure S8b), particularly in the content of α-helix and β-sheet. The A209C/R283C variant and the N364C/D418C variant showed high α-helix content and low β-sheet content. However, the sum of α-helix and β-sheet was similar to that of *Bb*PETase^CD^. For the P207C/D280C/N364C/D418C and A209C/R283C/N364C/D418C variants, increased random coil content was observed.

The wavelengths corresponding to the fluorescence peaks of the *Bb*PETase^CD^ and the variants were nearly identical (Figure S9a). No obvious difference was observed between the *Bb*PETase^CD^ and the N364C/D418C variant in the fluorescence spectra, the endogenous tryptophan fluorescence spectra, or the endogenous tyrosine fluorescence spectra (Figure S9). The other four variants showed a slight red shift in the endogenous tryptophan fluorescence spectra compared to *Bb*PETase^CD^ (Figure S9b). This indicated that the two pairs of disulfide bridges, P207C/D280C and A209C/R283C, had a significant effect on the structure of the enzyme. In the endogenous tyrosine fluorescence spectra (Figure S9c), compared to the *Bb*PETase^CD^, P207C/D280C variant showed a small red shift, and the left three variants showed blue shifts. Only the P207C/D280C variant had a lower *T_m_* value, most likely due to the negative effect of the P207C-D280C disulfide bridge on the structure and stability of *Bb*PETase^CD^.

## 3. Materials and Methods

### 3.1. Bacterial Strains, Plasmid, and Materials

*E*. *coli* JM109 served as the host strain for site-specific mutagenesis. *E*. *coli* Rosetta gami-B (DE3) (Weidi Biotech, Shanghai, China) was used to express various mutant enzymes. The gene for *Bb*PETase^CD^ was synthesized by Genewiz (Suzhou, China) and using pET-22b(+) plasmid as gene vector. DNA sequencing was performed by Genewiz (Suzhou, China). 5 mL His Trap^TM^ FF affinity chromatography column was purchased from GE Healthcare (9 × 15.7 mm, 90 μm, Marlborough, MA, USA). Fast pfu enzyme and DpnI enzyme were purchased from TransGen Biotech (Beijing, China). A plasmid extraction kit was purchased from Beyotime (Shanghai, China). A BCA kit was purchased from Dingguo Biotech (Tianjin, China). A sulfhydryl assay kit was purchased from Bestbio (Shanghai, China). The crystallinity of PET films was measured using DSC by Standard (Qingdao, China). Other reagents were purchased from Sangon Biotech (Shanghai, China), Heowns (Tianjin, China), or Meryer (Shanghai, China).

### 3.2. Rational Design of Disulfide Bridges

The wild-type *Bb*PETase^CD^ has two natural disulfide bridges, C333-C370 and C404-C424 [17]. To improve the thermal stability of the enzyme, we used DbD2 and the DSDBASE-MODIP program to predict mutation sites in the structure of *Bb*PETase^CD^ that could form disulfide bridges and then screened them using the conditions listed below. Because disulfide bridges that were too close together could have an adverse effect on the enzyme structure, mutation sites that were too close together (fewer than 10AAs in the primary structure) were removed at first [27]. Secondly, the preferential selection of mutation sites located in the flexible loop region may improve the thermal stability of the enzyme by increasing its local rigidity [18]. Meanwhile, predicted mutation sites should avoid the enzyme’s active site or sites related to substrate binding to avoid affecting enzyme activity. Finally, the variants were homology-modeled by the SWISS-MODEL program [24] and visualized by PyMOL (http://www.pymol.org, accessed on 26 November 2022) to see if they could correctly form disulfide bridges.

### 3.3. Site-Directed Mutagenesis

All disulfide-bridge mutations were introduced by polymerase chain reaction (PCR). After the PCR, the methylated template was removed with DpnI restriction endonuclease for 20 min at 37 °C. The concentrated PCR products were transformed into competent *E*. *coli* JM109 cells, and mutant plasmids were screened at 37 °C on Luria–Bertani (LB) agar plate medium containing 100 μg/mL sodium ampicillin. DNA sequencing was also used to identify the mutant plasmids. The correctly sequenced plasmid DNA was transformed into *E*. *coli* Rosetta gami-B (DE3) for protein expression. Table S1 shows the primers used for PCR, with underlines indicating the codons and anticodons corresponding to the mutation.

### 3.4. Expression and Purification

*E. coli* Rosetta gami-B (DE3) strains containing various mutant plasmids were incubated aerobically at 220 rpm and 37 °C in LB medium containing 100 μg/mL ampicillin sodium until the optical density at 600 nm (OD_600_) reached approximately 0.8–1.0 [17]. Isopropyl β-D-thiogalactoside (IPTG) was added to a final concentration of 0.5 mM to induce protein expression. After 24 h of protein expression at 16 °C, the bacteria were harvested by centrifugation at 4000 rpm for 30 min at 4 °C. The bacteria were resuspended in 25 mL of lysis buffer (30 mM imidazole, 50 mM Na_2_HPO_4_, and 100 mM NaCl buffer, pH 7.0) and lysed for 30 min using ultrasonication. After centrifugation at 10,000 rpm for 30 min at 4 °C, the supernatant was obtained and then filtered through a 0.45 μm filter membrane. The enzyme was purified in an AKTA-Basic chromatography system (GE Healthcare, Marlborough, MA, USA) using a 5 mL His Trap^TM^ FF affinity chromatography column. The column was first equilibrated with lysis buffer at a flow rate of 5 mL/min, washed with lysis buffer at a flow rate of 5 mL/min for 5 min after supernatant loading was completed, then eluted with elution buffer (500 mM imidazole, 50 mM Na_2_HPO_4_, and 100 mM NaCl buffer, pH 7.0) at a flow rate of 1 mL/min for 15 min. To remove imidazole, the purified protein solution was centrifuged at 6000 rpm for 30 min through a 10 kDa ultrafiltration tube. Finally, the enzyme concentration was determined using the BCA kit according to the instructions and diluted to the reaction concentration (5 μM). Sodium dodecyl SDS-PAGE 12% was used to determine the purity and the molecular weight.

### 3.5. Analysis of Disulfide Bridge of Enzyme

To determine the sulfhydryl content of sample, the sample was mixed with the working solution in the sulfhydryl assay kit according to the instructions, and the reaction was carried out at room temperature for 15 min, followed by testing of OD_412_. All measurements were conducted in triplicate (*n* = 3). The standard curve was obtained by fitting the OD_412_ to the concentration of L-cysteine using standard solution of different concentrations, which was then used for the calculation of sulfhydryl content. Thereafter, the enzyme (5 μM) was prepared using the working solution, followed by the determination of sulfhydryl content under nonreducing condition. Meanwhile, 50 mM tris(2-carboxyethyl) phosphine (TCEP-HCl) was added to another group of enzyme (5 μM) to convert the disulfide bridges in the enzymes to sulfhydryl, followed by the determination of sulfhydryl content under reducing condition. Thereafter, the proportion of disulfide bridges formed in the enzyme was determined by comparing the sulfhydryl content of the enzyme under nonreducing and reducing conditions.

### 3.6. Fluorescence and CD Spectrums

The fluorescence analysis was performed at room temperature on a fluorescence spectrometer (PerkinElmer, Waltham, MA, USA) using a quartz cuvette with a path length of 1.0 cm. It was excited at 280 nm, and the emission spectra were detected in the range of 250–450 nm with a slit width of 5.0 nm for both excitations and emissions. The excitation wavelengths were set to 250–330 nm and the scanning intervals (Δλ) were set to 15 and 60 nm to obtain their synchronous fluorescence spectra, where Δλ at 15 and 60 nm represent the fluorescence spectra of endogenous tyrosine (Tyr, Y) and tryptophan (Trp, W), respectively.

The CD spectrum was tested by a Jasco J-810 circular dichroism spectrometer (Jasco, Tokyo, Japan) using a quartz cuvette with a path length of 1.0 mm. Continuous scanning was measured at 30 °C in the wavelength range of 190–260 nm with a data pitch of 0.1 nm, a response time of 1.0 s, a bandwidth of 2.0 nm, and a scanning speed of 100 nm/min. The secondary structure of the enzyme was analyzed by Dichroweb (http://dichroweb.cryst.bbk.ac.uk/, accessed on 16 February 2023) on-line analysis [28,29]. To obtain the *T_m_* value of *Bb*PETase^CD^ and its variants, the thermal denaturation curves of enzymes by CD detection were used. Specifically CD signal at 222 nm was examined as a function of temperature. The data was then fitted with a Boltzmann curve, and the *T_m_* value was the temperature corresponding to the fitted curve’s midpoint.

### 3.7. Enzyme Activity Using BHET as Substrate

The enzyme activity was examined at 30 °C, 40 °C, and 50 °C with the small molecule BHET as the substrate. For the reaction, 910 μL of neutral reaction buffer (80 mM Na_2_HPO_4_, 40 mM NaCl, pH 7.0) or 910 μL of basic reaction buffer (50 mM Gly-NaOH, pH 9.0) was used. Then, 10 μL of enzyme solution (5 μM) and 80 μL of BHET solution (2.5 g/L) were added, so the final concentration of enzyme was 50 nM while the final concentration of BHET was 200 μg/mL. The reaction was carried out for 30 min. At the end of the reaction, an equal volume of reaction stop buffer (160 mM NaH_2_PO_4_, 20% *v*/*v* DMSO, pH 2.5) was rapidly added, followed by a 10 min water bath at 85 °C. After 10 min of centrifugation at 12,000 rpm, the samples were passed through a 0.22 μm filter membrane and the products were analyzed using high-performance liquid chromatography (HPLC). All measurements were conducted in triplicate (*n* = 3).

### 3.8. Thermal Stability of the Enzyme

To test the activity retention of enzyme after incubation at high temperature, we mixed the enzymes with basic reaction buffer, followed by incubation. Herein, 50 °C and 40 °C (temperature corresponding to long-term degradations of PET films) were used. For 50 °C, incubation time of 1 h, 2 h, 4 h, and 6 h was used. For 40 °C, incubation times of 1 h, 2 h, 4 h, 6 h, 9 h, 12 h, and 24 h were used. After incubation, the enzyme was placed in ice water for 10 min and 80 μL of BHET solution was then added. The reaction was carried out at 50 °C in a water bath for 30 min. The protocol of terminating and analyzing the reaction was the same as that described in Section 3.7. All measurements were conducted in triplicate (*n* = 3).

### 3.9. Enzyme Activity for PET Film Degradation

A mineral water bottle (crystallinity of 32.3%, NongFu Spring, Tianjin, China) was used as substrate. It was washed with 1% *w*/*v* SDS solution, ethanol, and deionized water, and was cut into films with a diameter of 6 mm. Each 2 mL centrifuge tube received two PET films, 300 μL basic reaction buffer (pH 9.0), and 12.5 μL enzyme for 6 days. Reaction temperature of 30 °C and 40 °C was used. Meanwhile, 8 sets of reactions were supplemented at 40 °C for 14 days. The protocol of terminating and analyzing the reaction was the same as that described in Section 3.7. Following the reaction, the PET was washed using the method described above. Traces of enzyme erosion on the PET surface were observed by field-emission scanning electron microscopy Apreo S LoVac (FEI, Hillsboro, OR, USA). All measurements were conducted in triplicate (*n* = 3).

### 3.10. HPLC Analysis

HPLC analysis of aromatic products was performed on an Agilent 1100 series LC system equipped with a Welch Ultimate XB-C18 column (4.6 × 250 mm, 5 μm, Welch Materials, Shanghai, China) at a controlled column temperature of 25 °C. The mobile phase contained 70% (*v*/*v*) distilled water, 20% (*v*/*v*) acetonitrile, and 10% (*v*/*v*) formic acid at a flow rate of 1.0 mL/min. The aromatic products were detected by absorbance at 254 nm and identified by the retention time of the standard compounds [30]. A calibration curve consisting of the absorption peak area and the concentration of the standard solution was used to calculate the concentration of each product. The calibration curve had an R-squared value of at least 0.999.

## 4. Conclusions

Rational introduction of disulfide bridges in *Bb*PETase^CD^ was performed through computer-aided rational design. P207C/D280C, A209C/R283C, N364C/D418C, P207C/D280C/N364C/D418C, and A209C/R283C/N364C/D418C variants were obtained. Among them, the N364C/D418C variant showed the most significant enhancement of the hydrolytic activity of PET films to about 11-fold that of *Bb*PETase^CD^ and obtained an increase in the *T_m_* value of 14.8 °C. The additional disulfide bridge could significantly improve the structural rigidity of *Bb*PETase^CD^, which in turn increased its stability and activity. The residual activity of the N364C/D418C variant in this study was not significantly higher than that of the other variants after thermal incubation, whereas its *T_m_* value was significantly higher than that of the other variants, indicating that the kinetic and thermodynamic stability of this variant did not exactly match. Usually, high kinetic stability confers resistance to enzyme inactivation at high temperatures, while high thermodynamic stability is manifested by the higher free energy of enzyme unfolding, but the two are determined by different molecular processes and therefore do not always correspond exactly [31]. The remaining three variants showed no significant improvement in activity and stability, except for the P207C/D280C/N364C/D418C variant, which showed increased activity. The N364C/D418C mutation was closer to the natural disulfide bond and the active site of the enzyme than the other two mutations. This resulted in a significant increase in the *T_m_* value of the enzyme by enhancing the regional structural rigidity of the enzyme [26], and we hypothesize that the introduction of the N364C/D418C mutation changed the structure of the enzyme, expanding its active site pocket and increasing its activity. In addition, the expression levels of the P207C/D280C, A209C/R283C, P207C/D280C/N364C/D418C and A209C/R283C/N364C/D418C variants were extremely low. We speculated that these four variants contained mutating points close to the *N*-terminal of the enzyme, which made the enzyme prone to misfolding and low expression during protein expression. It has been reported that inefficient disulfide bonds can have a negative impact on enzyme activity and/or stability [32]. This may provide some reference for schemes to improve enzyme stability through rational design of disulfide bridges, and mutations near the *N*-terminal of proteins should be avoided for *Bb*PETase^CD^.

## Figures and Tables

**Table 1:** Proportion of disulfide bridge of *Bb*PETase^CD^ and its variants.

Enzyme	Sulfhydryl Content under Nonreducing Condition (μM)	Sulfhydryl Content under Reducing Condition (μM)	The Proportion of Disulfide Bridge (%)
*Bb*PETase^CD^	1.2	20.1	93.8
P207C/D280C	4.7	26.5	82.2
A209C/R283C	6.2	26.2	76.3
N364C/D418C	3.3	30.6	89.3
P207C/D280C/N364C/D418C	6.1	37.7	83.8
A209C/R283C/N364C/D418C	5.5	39.4	86.2

## Data Availability

Data are available on request from the authors.

## Supplementary Materials

The following supporting information can be downloaded at https://www.mdpi.com/article/10.3390/molecules28083528/s1. Table S1. Information on primers used for targeted mutagenesis; Table S2. Expression of *Bb*PETase^CD^ and its variants; Figure S1. The distance between P207 and A209 and the distance between D280 and R283. Figure S2. Location of disulfide bridges in variants of *Bb*PETase^CD^; Figure S3. Affinity purification of *Bb*PETase^CD^ and its variants using nickel ion affinity chromatography; Figure S4. SDS-PAGE electrophoresis of *Bb*PETase^CD^ and its variants in nonreducing condition; Figure S5. Melting curves of *Bb*PETase^CD^ and its variants; Figure S6. DSC diagram of Nongfu Spring mineral water bottle; Figure S7. SEM of PET film after 14 days of incubation with *Bb*PETase^CD^ and its variants; Figure S8. CD spectra of *Bb*PETase^CD^ and its variants with secondary structure content; Figure S9. Fluorescence spectra of *Bb*PETase^CD^ and its variants.

## Sample Availability

Samples of the compounds are available from the authors.
