# Supplementary material for: Rational Design of Disulfide Bridges in BbPETaseCD for Enhancing the Enzymatic Performance in PET Degradation
Source: Molecules. 2023 Apr 17;28(8):3528. doi: 10.3390/molecules28083528 (PMC10146679; doi:10.3390/molecules28083528)
Supplement: Supplementary file 1 [file molecules-28-03528-s001.zip › molecules-2326480-supplementary.pdf]

Supplementary Materials

Rational Design of Disulfide Bridges in *Bb*PETase^{CD} for Enhancing the Enzymatic Performance in PET Degradation

Dongjian Huang [1], Lin Zhang [1,2] and Yan Sun [1,2,*]

1 Department of Biochemical Engineering, School of Chemical Engineering and Technology, Tianjin University, Tianjin 300350, China; huangdongjian@tju.edu.cn (D.H.); linzhang@tju.edu.cn (L.Z.)
2 Key Laboratory of Systems Bioengineering and Frontiers Science Center for Synthetic Biology (Ministry of Education), Tianjin University, Tianjin 300350, China
* Correspondence: ysun@tju.edu.cn

Table S1. Information on primers used for targeted mutagenesis

Primer	Primer sequence (5' to 3')
Forward-P207C	CGTGACGGGC**TGC**TTTGCGGCGGTGGCGGTGGTGCC
Reverse-P207C	CCGCCGCAAA**GCA**GCCCGTCACGTTGGTCGGATGAT
Forward-D280C	TCGCAAAGTG**TGT**CCGAACCGCCTGGGCGTGATGGG
Reverse-D280C	GGCGGTTCGG**ACA**CACTTTGCGATAAATCGGATGGC
Forward-A209C	GGGCCCGTTT**TGC**GCGGTGGCGGTGGTGCCGGGCTA
Reverse-A209C	CCGCCACCGC**GCA**AAACGGGCCCGTCACGTTGGTCG
Forward-R283C	GGATCCGAAC**TGC**CTGGGCGTGATGGGCTGGAGCAT
Reverse-R283C	TCACGCCCAG**GCA**GTTCGGATCCACTTTGCGATAAA
Forward-N364C	TCTGGAAATG**TGC**AACGGCAGCCATAGCTGCGCGAA
Reverse-N364C	GGCTGCCGTT**GCA**CATTTCCAGATACGCTTTTTTGG
Forward-D418C	GACCGCGATT**TGT**GAATATCGCGAAAACTGCCCGTA
Reverse-D418C	CGCGATATTC**ACA**AATCGCGGTCAGGCTCAGATCCG

Note: where the underline indicates the codon or anticodon in the primer.

Table S2. Expression of *Bb*PETase^{CD} and its variants

Enzyme	Protein expression (mg/L)
*Bb*PETase^{CD}	24.04
P207C/D280C	1.22
A209C/R283C	1.69
N364C/D418C	27.77
P207C/D280C/N364C/D418C	0.91
A209C/R283C/N364C/D418C	1.09

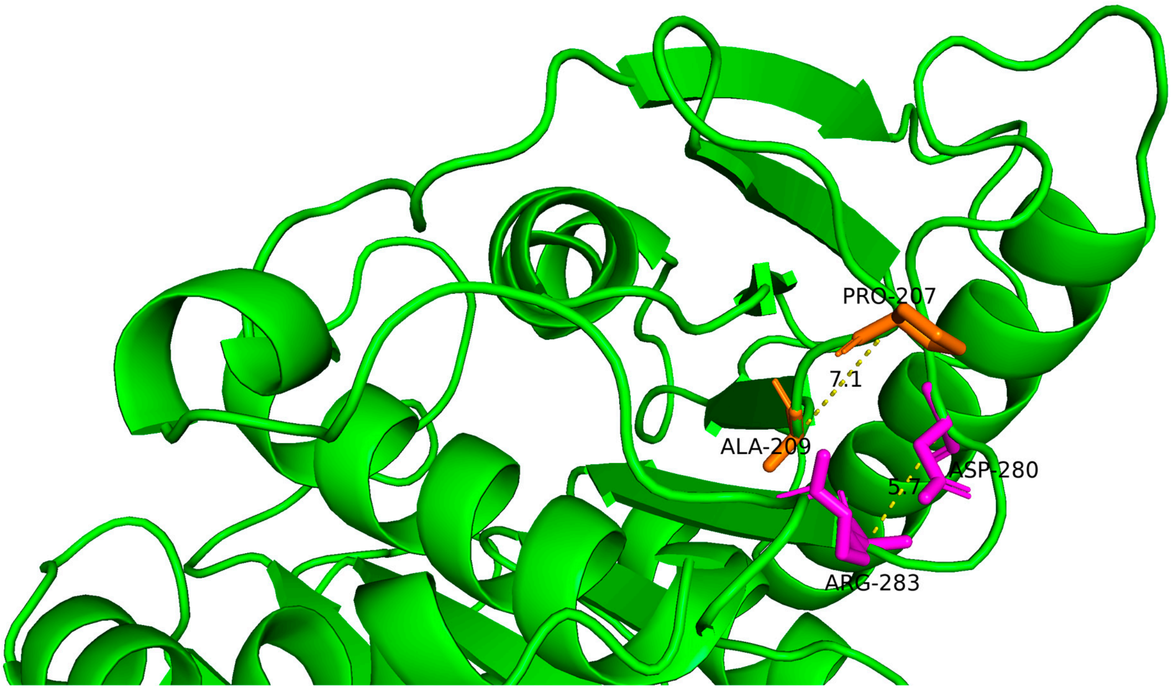

Figure S1. Diagram of the distance between P207 and A209 and the distance between D280 and R283 in the *Bb*PETase^{CD} structure (PDB ID: 7CWQ).

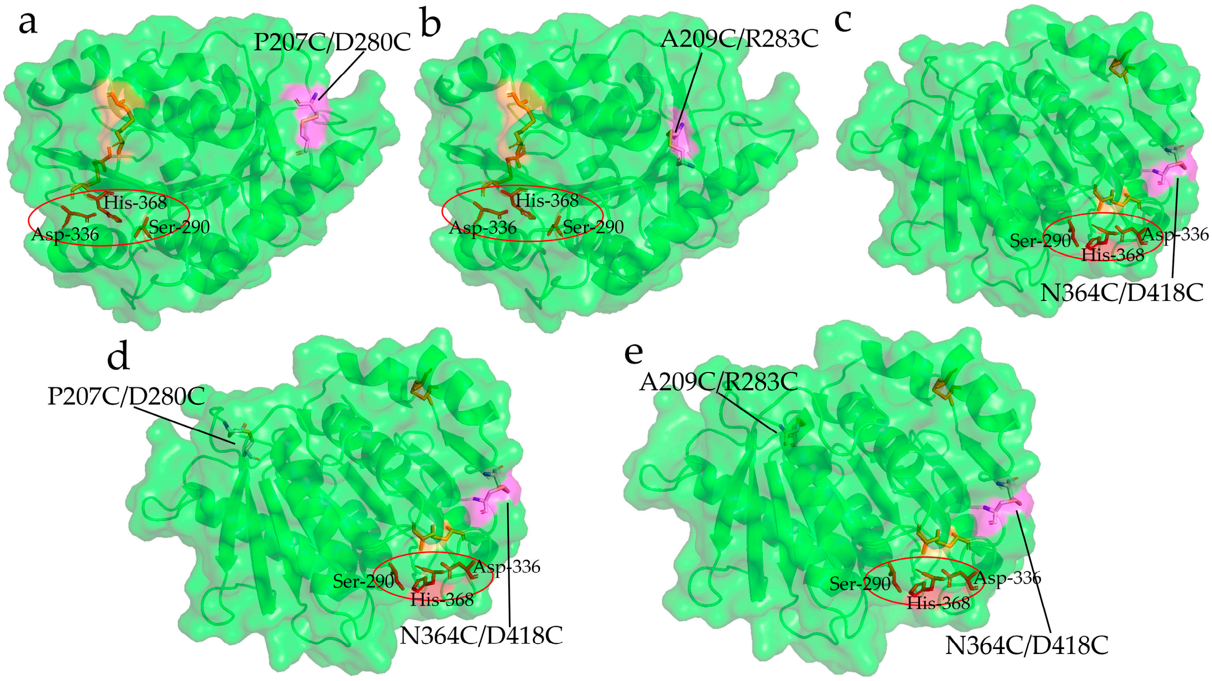

Figure S2. Location of disulfide bridges in variants of *Bb*PETase^{CD}, where the natural disulfide bridges in the enzyme are marked in orange and the mutation-introduced disulfide bridges are marked in purple. The amino acid residues marked in red are the catalytic active sites of *Bb*PETase^{CD}. (a) P207/D280C variant, (b) A209C/R283C variant, (c) N364C/D418C variant, (d) P207/D280C/N364C/D418C variant, (e) A209C/R283C/N364C/D418C variant.

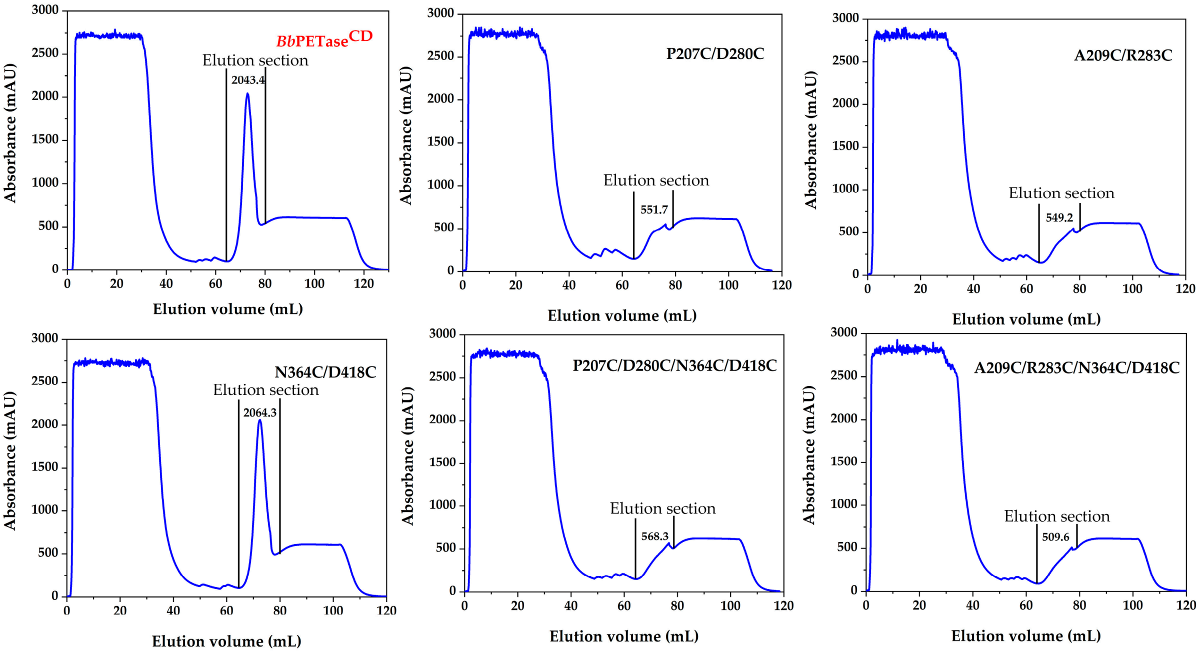

Figure S3. Affinity purification of *Bb*PETase^{CD} and its variants using Nickel ion affinity chromatography, where the elution section is the gradient elution stage and the numbers indicate the absorbance of the eluted peak.

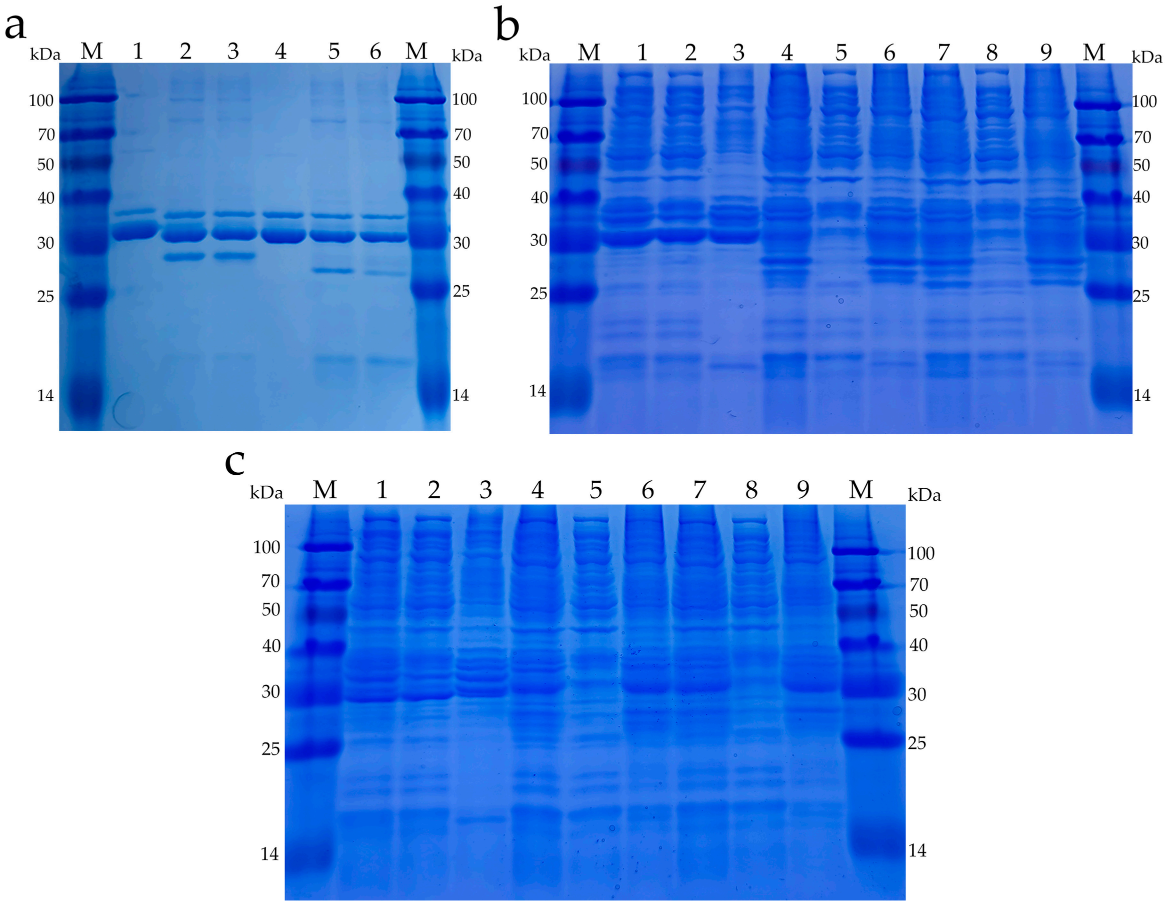

Figure S4. (a) SDS-PAGE of *Bb*PETase^{CD} and its variants in nonreducing condition. M: Marker, Lane1: *Bb*PETase^{CD}; Lane2: P207/D280C variant; Lane3: A209C/R283C variant; Lane4: N364C/D418C variant; Lane5: P207/D280C/N364C/D418C variant; Lane6: A209C/R283C/N364C/D418C variant. The loading concentration of each gel lane is 0.2175mg/ml, and the loading volume is 10μl. (b) Lane1: bacterial lysate of *Bb*PETase^{CD}; Lane2: supernatant of *Bb*PETase^{CD}; Lane3: precipitate of *Bb*PETase^{CD}; Lane4: bacterial lysate of P207/D280C variant; Lane5: supernatant of P207/D280C variant; Lane6: precipitate of P207/D280C variant.

Lane7: bacterial lysate of A209C/R283C variant; Lane8: supernatant of A209C/R283C variant; Lane9: precipitate of A209C/R283C variant. (c) Lane1: bacterial lysate of N364C/D418C variant; Lane2: supernatant of N364C/D418C variant; Lane3: precipitate of N364C/D418C variant; Lane4: bacterial lysate of P207/D280C/N364C/D418C variant; Lane5: supernatant of P207/D280C/N364C/D418C variant; Lane6: precipitate of P207/D280C/N364C/D418C variant.

Lane7: bacterial lysate of A209C/R283C/N364C/D418C; Lane8: supernatant of A209C/R283C/N364C/D418C variant; Lane9: precipitate of A209C/R283C/N364C/D418C variant.

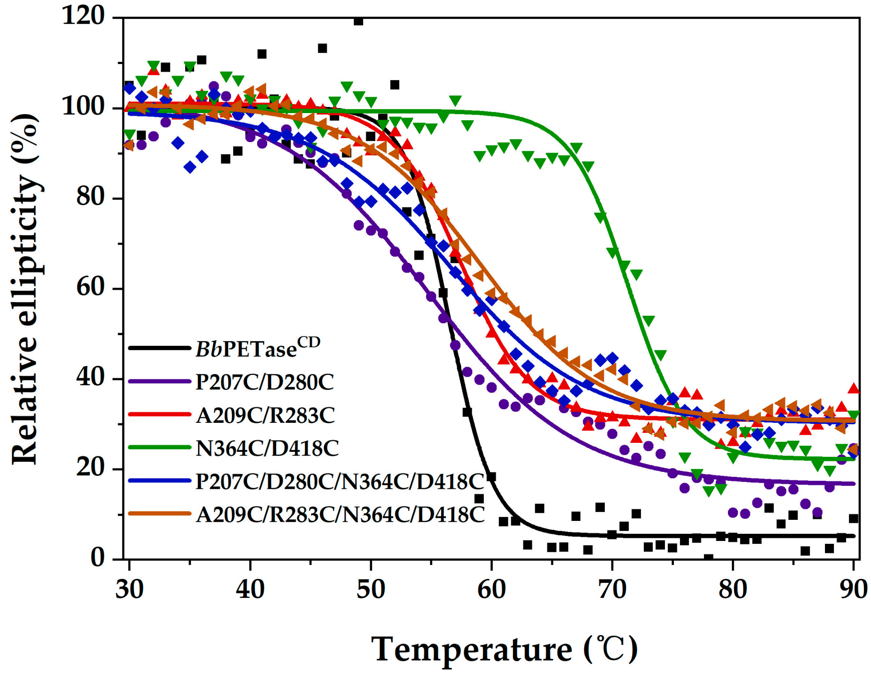

Figure S5. Melting curves of *Bb*PETase^{CD} and its variants.

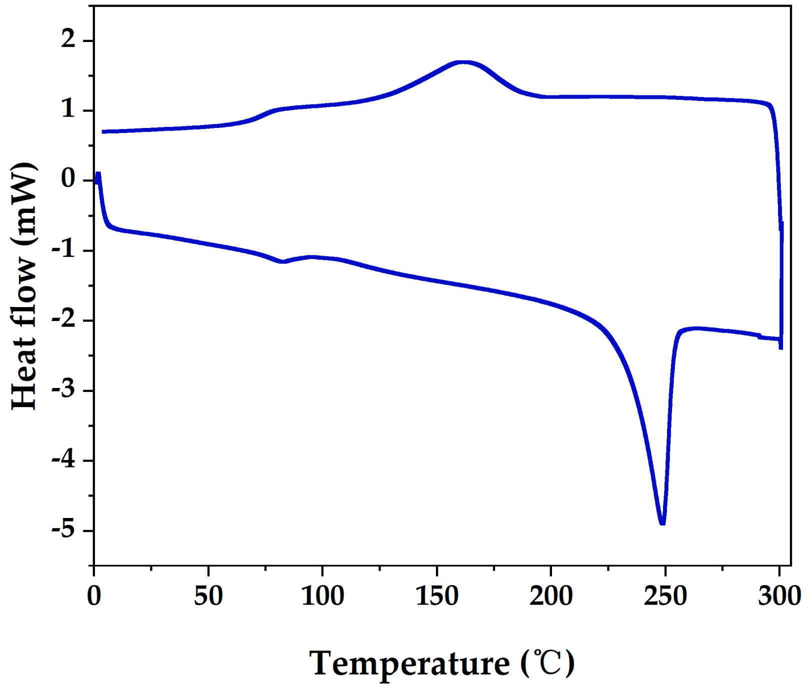

Figure S6. DSC diagram of Nongfu Spring mineral water bottle.

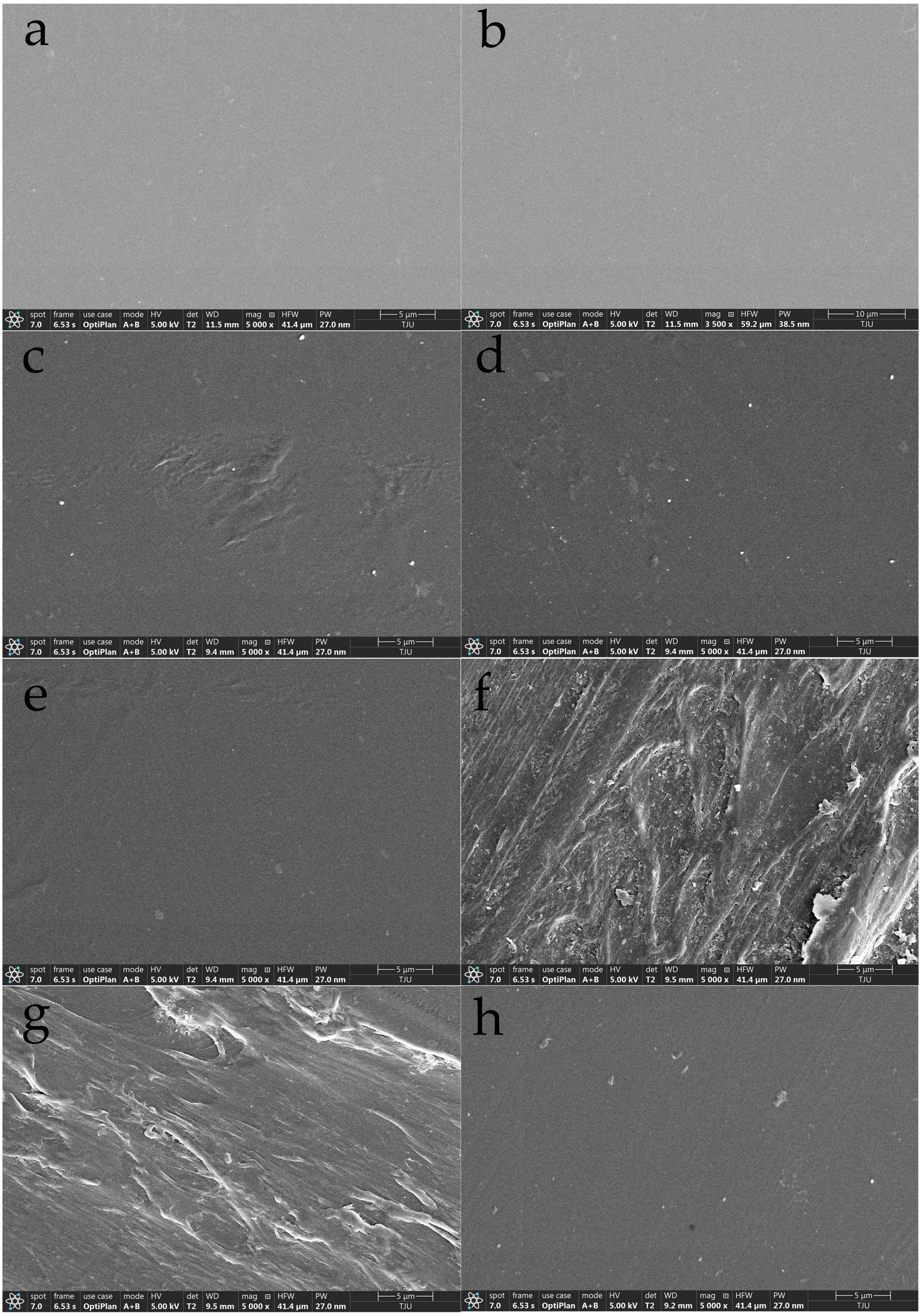

Figure S7. SEM pictures of PET film after 14 days of incubation with *Bb*PETase^{CD} and its variants. (a) and (b) are SEM pictures of unreacted PET film at different magnifications, and the remaining SEM pictures are PET film after incubation with (c) *Bb*PETase^{CD}, (d) P207/D280C variant, (e) A209C/R283C variant, (f) N364C/D418C variant, (g) P207/D280C/N364C/D418C variant, (h) A209C/R283C/N364C/D418C variant.

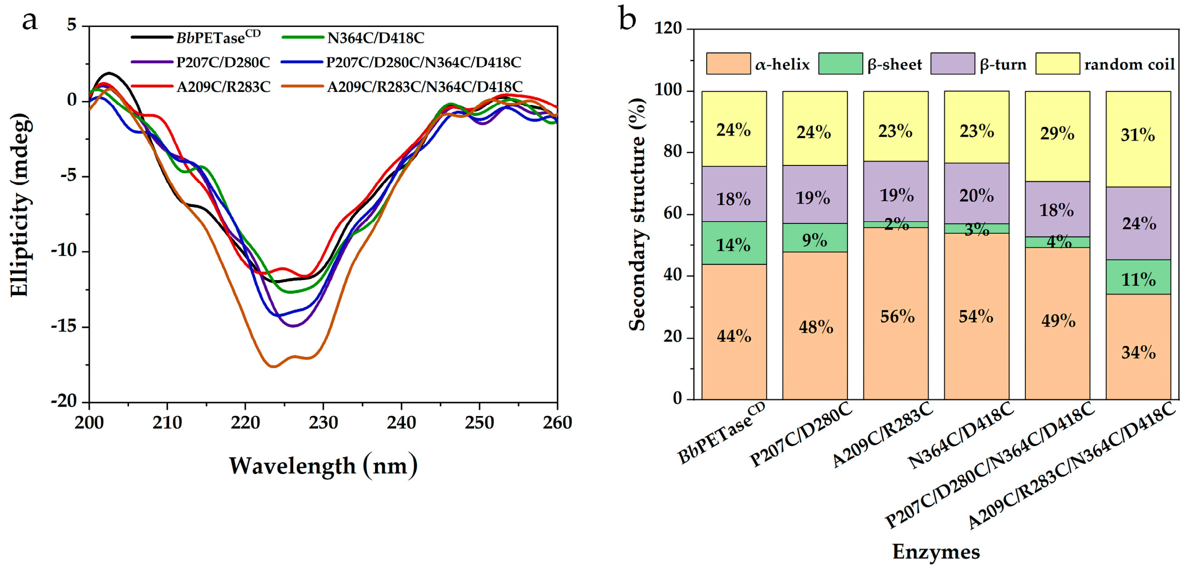

Figure S8. CD spectra of *Bb*PETase^{CD} and its variants (a) with secondary structure content (b).

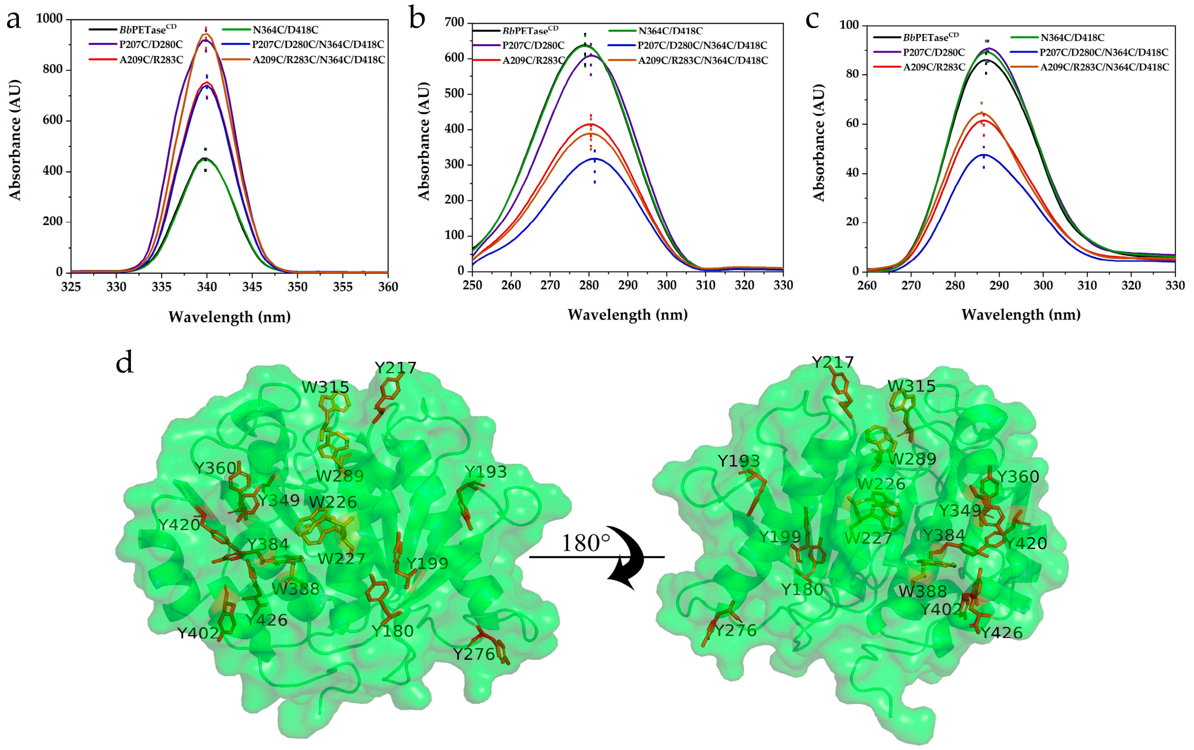

Figure S9. Fluorescence spectra (a), endogenous tryptophan fluorescence spectra (b) and endogenous tyrosine fluorescence spectra (c) of *Bb*PETase^{CD} and its variants, (d) position of tryptophan (orange) and tyrosine (red) in *Bb*PETase^{CD} (PDB ID: 7CWQ).
